# Dysbiotic Vaginal Microbiota Induces Preterm Birth Cascade via Pathogenic Molecules in the Vagina

**DOI:** 10.3390/metabo14010045

**Published:** 2024-01-11

**Authors:** AbuZar Ansari, Young-Ah You, Gain Lee, Soo Min Kim, Sun Wha Park, Young Min Hur, Young Ju Kim

**Affiliations:** 1 Department of Obstetrics and Gynecology and Ewha Medical Research Institute, Ewha Womans University, Seoul 07985, Republic of Korea; yayou@ewha.ac.kr (Y.-A.Y.); loveleee0102@gmail.com (G.L.); zeus_0218@naver.com (S.M.K.); clarrissa15@gmail.com (S.W.P.); k0507hym@hanmail.net (Y.M.H.); 2 Graduate Program in System Health Science and Engineering, Ewha Womans University, Seoul 07984, Republic of Korea

**Keywords:** cervicovaginal fluid, microbiota, metabolite, cytokine, preterm birth

## Abstract

Dysbiotic vaginal microbiota (DVM) disturb the vaginal environment, including pH, metabolite, protein, and cytokine profiles. This study investigated the impact of DVM on the vaginal environment in 40 Korean pregnant women and identified predictable biomarkers of birth outcomes. Cervicovaginal fluid (CVF) samples were collected in the third trimester using vaginal swabs, examined for pH, and stored at −80 °C for further analysis. The samples were grouped as full-term (FTB, n = 20) and preterm (PTB, n = 20) births. The microbiota was profiled in the V1–V9 regions. The levels of targeted metabolites, TLR-4, and cytokines were determined. The pH of CVF from PTB (>4.5) was significantly higher than that of the CVF from FTB (>3.5) (*p* < 0.05). Neonatal gestational age at delivery, birth weight, and Apgar score differed significantly between groups. The relative abundances of beneficial *Lactobacillus* spp., such as *Lactobacillus gasseri*, *Lactobacillus jensenii*, and *Bifidobacterium*, were higher in FTB, whereas those of pathogenic *Enterococcus faecalis*, *Staphylococcus*, *Prevotella*, *Ureaplasma parvum*, and *Corynebacterium* spp. were higher in PTB. Acetate, methanol, TLR-4, and TNF-α levels were negatively correlated with gestational age at delivery and birth weight. Moreover, ethanol, methanol, TLR-4, IL-6, IL-1β, and TNF-α levels were positively correlated with succinate, acetate, acetoacetate, formate, and ammonia. Overall, DVM induces preterm birth via pathogenic molecules in the vagina.

## 1. Introduction

Recent studies have revealed that over 13.4 million neonates are born preterm (birth before 37 weeks of gestation), and the incidence of preterm birth continues to increase globally, including in South Korea [1]. Up to 40% of preterm births (PTBs) are associated with inflammation caused by intrauterine infections [2], which generally originate from the urogenital tract (vaginal route) or the hematogenous route through placental translocation from the digestive tract (gut route) [3,4]. Dysbiotic vaginal microbiota (DVM) cause urogenital infections, which are often followed by an abundance of gram-negative bacteria [5]. Basically, DVM is a shift with a decrease in beneficial *Lactobacillus* spp. and an increase in other microbiota in the vagina, which also leads to inflammation during pregnancy and may induce PTB [6]. Inflammation is the most common molecular mechanism of PTB through the TLR signaling pathway [7].

Many factors in PTB indicate a disturbed vaginal environment, such as a high pH (>4.5), which is the first predictive marker of a disturbed vaginal environment [8]. Sexual transmission and pathogenic microbial invasion are also important indications of a disturbed vaginal environment and indicate bacterial vaginosis (BV) [9]. BV is often associated with a decrease in *Lactobacillus* spp. and an increase in pathogenic microbiota, such as *Gardnerella vaginalis*, *Prevotella*, *Enterococcus*, *Staphylococcus*, *Ureaplasma* spp., and other anaerobes [9]. Disturbed vaginal environments are relatively rich in anaerobic bacteria, such as *Prevotella*, *Dialister*, *Atopovium*, *Gardnerella*, *Megasphaera*, *Peptoniphilus*, *Sneatia*, *Eggertella*, *Aerococcus*, *Finegoldia*, and *Mobiluncus*, and have a higher pH (>4.5) than an environment with *Lactobacillus* spp., which maintain a lower pH (4.0 to <4.5) [10]. A high diversity of anaerobic microbial pathogens, as observed in BV, is associated with an increased risk of vaginal infection and inflammation, which result in PTB [11]. *Lactobacillus* spp. are considered beneficial vaginal microbes that protect against infection and invasion by pathogenic microbes [12,13]. The dominance of *Lactobacillus crispatus*, *Lactobacillus iners*, *Lactobacillus jensenii*, and *Lactobacillus gasseri* is considered a hallmark of a healthy vaginal environment and is associated with full-term birth (FTB) [14].

DVM may have a direct effect on the availability of microbiota-generated metabolites and birth outcomes. Lactic acid, a metabolite produced by dominant *Lactobacillus* spp., inhibits pathogen invasion and inhibition [15]. L-lactic acid has anti-inflammatory properties that inhibit the production of proinflammatory cytokines and chemokines induced by Toll-like receptors (TLRs) in vaginal epithelial cells at low pH (4.0 to <4.5) [15]. Furthermore, high levels of formate, succinate, and acetate affect proinflammatory cytokine production and birth outcomes [16,17].

DVM affects the vaginal health of millions of women; therefore, understanding the association between vaginal microbiota, metabolites, and cytokines in PTB is critical. Our previous studies on the relationship between a dysbiotic vaginal environment and PTB produced conflicting findings regarding whether the vaginal microbiome, metabolites, and cytokines can influence the risk of PTB [4,18]. In the present study, we investigated the pathophysiological aspect of DVM that induce the PTB via pathogenic molecules like metabolites and inflammatory factors in the vaginal environment.

## 2. Materials and Methods

### 2.1. Study Subjects and Cervicovaginal Fluid Sampling

Pregnant women were enrolled based on their clinical profiles with consent. Forty pregnant women were selected for this case-control study based on cervicovaginal fluid (CVF) collection in the third trimester of gestation. CVF samples were collected from pregnant women using vaginal swabs and stored at −80 °C for further analysis. After delivery, the CVF samples were grouped as FTB (n = 20) and PTB (n = 20). The collected CVF samples were centrifuged at 5000 rpm for 20 min at 4 °C. After centrifugation, bacterial DNA was isolated from the pellet for microbiota profiling, and the supernatant was used for metabolite and cytokine profiling. The study participants were enrolled from January 2021 to April 2023 at Ewha Womans University, Mokdong Hospital, Republic of Korea (Ethical Research Committee approval number: EUMC 2020-07-032). This study was conducted in accordance with the ethical principles of the Declaration of Helsinki. All participants provided written informed consent.

### 2.2. pH Determination in Cervicovaginal Fluid Samples

At the time of CVF sample collection, pH was measured immediately using a pH strip (Merck Millipore, Darmstadt, Germany). Twenty microliters of the CVF samples were placed on the pH strip to determine the pH level at room temperature, and the color was matched with the pH strip indicator.

### 2.3. Microbiota Analysis in Cervicovaginal Fluid Samples

We conducted a strain-level analysis of the microbiome using the region from V1 to V9. Next-generation sequencing was outsourced to Molecular Diagnostics Korea Inc. (Seoul, Republic of Korea). Briefly, DNA was isolated from CVF pellets using a microbial DNA extraction kit (Qiagen, Germantown, MD, USA). The quality and quantity of the extracted DNA were determined using a NanoDrop Microvolume Spectrophotometer (Thermo Fisher, Waltham, MA, USA). Subsequently, the DNA was amplified for library construction using the SHORELINE BIOME Kit on a polymerase chain reaction (PCR) machine (Bio-Rad, Hercules, CA, USA). A mixture of 10 μL of 2X PCR premix and 10 μL of Shoreline Biome Lysis Mix was added to each barcoded primer tube and capped; the 20 μL reaction was mixed thoroughly by gentle vortexing and spun down. The tube was then transferred to the PCR machine. The PCR conditions were as follows: denaturation at 95 °C for 3 min, amplification at 95 °C for 30 s, 59 °C for 45 s, 72 °C for 2 min, and final extension at 72 °C for 3 min. The amplified product of 1.5 μL diluted with 5 μL gel loading dye was electrophoresed on 1% agarose gel in TBE at 15V for approximately 45 min with a DNA ladder. A band of ~2500 bp was observed using a gel documentation system (Bio-Rad). After band confirmation, the remaining samples were sent to Molecular Diagnostics Korea Inc. for microbiota profiling.

### 2.4. Measurement of Metabolites in Cervicovaginal Fluid Samples

Target metabolites were selected based on our previous study [19]. We targeted beneficial (L-lactate) and pathogenic (trimethylamine N-oxide (TMAO), formate, succinate, formaldehyde, acetoacetate, ammonia, acetate, ethanol, methanol) metabolites. Levels of lactate, TMAO, formate, succinate, formaldehyde, acetoacetate, and ammonia were measured in the supernatant of the CVF sample using the BM-LAV-100 (Biomax Co., Ltd., Guri-si, Republic of Korea), MBS7269386 (MyBioSource, San Diego, CA, USA), ab111748 (abcam, Shanghai, China), ab204718 (abcam, Shanghai, China), MAK131 (Merck, Rahway, NJ, USA), ab180875 (Shanghai, China), AA0100 (Merck, Rahway, NJ, USA), BM-ETH-100 (Biomax Co., Ltd., Guri-si, Republic of Korea), and ab241033 (abcam, Shanghai, China) assay kits, respectively, according to the manufacturer’s instructions.

### 2.5. Measurement of Protein Receptors in Cervicovaginal Fluid Samples

TLR-4, the most inflammatory transmembrane protein, is a pattern recognition receptor (PRR) that induces the PTB cascade [20]. TLR-4 was measured in the supernatant of CVF samples using an enzyme-linked immunosorbent assay (ELISA) kit (KTE60314; Abbkine, Inc., Atlanta, GA, USA) according to the manufacturer’s instructions.

### 2.6. Measurement of Cytokines in Cervicovaginal Fluid Samples

Based on our previous findings, we targeted proinflammatory cytokines; chemokine CCL3 (also known as macrophage inflammatory protein 1 alpha), interleukins (IL; IL-6, IL-7, and IL-1β), and tumor necrosis factor alpha (TNF-α) [21]. The cytokines CCL3 (KET6002; Abbkine Inc.), IL-6 (KET6017; Abbkine, Inc.), IL-7 (MBS453414; MyBioSource), IL-1β (KET6013; Abbkine, Inc.), and TNF-α (ab181421; Abcam, Shanghai, China) were measured in the supernatants of CVF samples via ELISA according to the manufacturer’s instructions. The intra-assay coefficient of variation (CV) was <10% and the inter-assay CV was <12%.

### 2.7. Statistical Analysis

Statistical analyses were performed using Student’s *t*-test, and *p* < 0.05 was considered statistically significant. We performed Spearman correlation analysis for the biophysical and biochemical variables of pregnant women and neonates, and the positive and negative correlations were determined based on the coefficient of correlation (r value); *p* < 0.05 was considered statistically significant.

## 3. Results

### 3.1. Study Participant Demographics

After delivery, the 40 women included in the study were grouped into two groups: FTB (n = 20) and PTB (n = 20). The pH levels of CVF samples were measured at the time of sample collection. The measured pH of the PTB (>4.5) group was significantly higher than that of the FTB (>3.5) group (*p* < 0.002). No significant differences were observed between the FTB and PTB groups in terms of age, body mass index, gestational age, or cervical length. However, significant differences were observed in gestational age at delivery, body weight, and 1 min and 5 min Apgar scores (*p* < 0.05) (Table 1).

### 3.2. Microbiota Analysis of Cervicovaginal Fluid Samples

**Figure 1:**
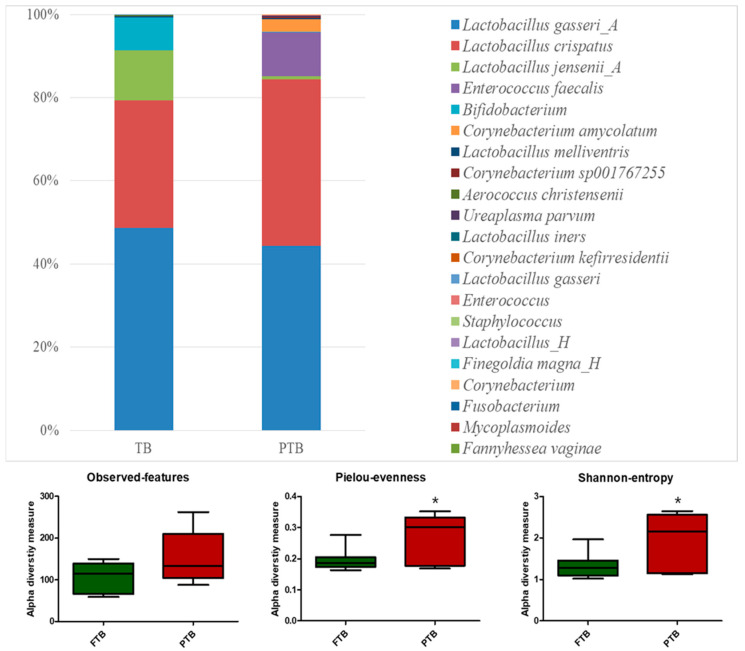
Microbiota analysis in cervicovaginal fluid (CVF) samples. Microbiota profiled in CVF samples of full-term birth (FTB, n = 7) and preterm birth (PTB, n = 7) groups via next-generation sequencing of the V1–V9 region. Relative abundance between FTB and PTB groups, and alpha diversity were calculated. Data are presented as the mean ± standard deviation, and *p* < 0.05 was considered sta-tistically significant; * *p* < 0.05.

Microbiota profiling of the CVF samples of FTB (n = 7) and PTB (n = 7) groups was performed via next-generation sequencing of the V1–V9 region. The complete microbiota profile is shown in Supplementary Figure S1. The relative abundances of beneficial *Lactobacillus jensenii_A* (*p* < 0.0001) and *Bifidobacterium* (*p <* 0.0001) were significantly higher in FTB, whereas that of pathogenic *Enterococcus faecalis* (*p <* 0.0001) was significantly higher in the PTB group. In addition, we observed a high relative abundance of *Corynebacterium* spp. in PTB compared to FTB. Diversity analysis of the microbiota showed a significantly higher Pielou evenness and Shannon entropy in PTB than in FTB, even though the observed features were significant (Figure 1, *p* < 0.05).

### 3.3. Metabolite Analysis of Cervicovaginal Fluid Samples

**Figure 2:**
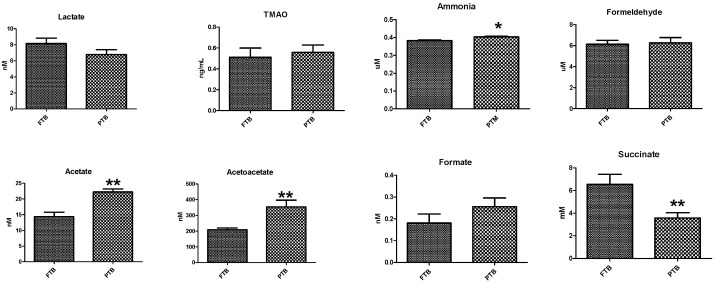
Metabolite analysis in cervicovaginal fluid (CVF) samples. Metabolites measured in CVF samples of full-term birth (FTB, n = 20) and preterm birth (PTB, n = 20) groups using the assay kits. Data are presented as the mean ± standard deviation, and *p* < 0.05 was considered statistically significant; * *p* < 0.05, ** *p* < 0.01.

Eight metabolites (L-lactate, TMAO, ammonia, formaldehyde, acetate, acetoacetate, formate, succinate, and acetate) were measured in the CVF samples. L-lactate levels were higher in the FTB group than in the PTB group, but the difference was not significant. The levels of four metabolites (ammonia, acetate, acetoacetate, and succinate) were significantly higher in PTB (*p* < 0.05) than in FTB. The levels of the other two metabolites (TMAO and formate) were higher in the PTB group than in the FTB group, but the difference was not significant (Figure 2). In addition, we determined the level of ethanol and methanol in CVF samples, and the levels were higher in PTB (*p* < 0.05) than in FTB (Supplementary Figure S2).

### 3.4. Analysis of Inflammatory Markers in Cervicovaginal Fluid Samples

**Figure 3:**
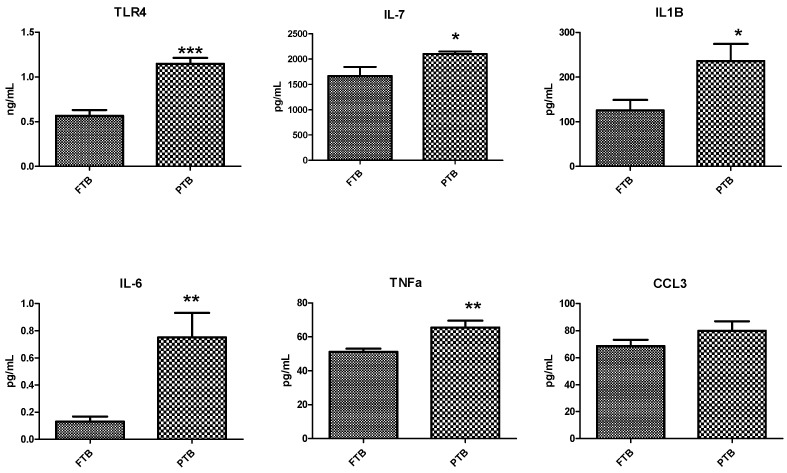
Analysis of inflammatory markers in cervicovaginal fluid (CVF) samples. Inflammatory markers measured in CVF samples of full-term birth (FTB, n = 20) and preterm birth (PTB, n = 20) using enzyme-linked immunosorbent assay (ELISA). Levels of TLR4, IL6, IL7, IL1β, TNFα, and CCL3. Data are presented as the mean ± standard deviation, and *p* < 0.05 was considered statistically significant. * *p* < 0.05, ** *p* < 0.01, *** *p* < 0.001.

In total, six targeted inflammatory markers were analyzed using appropriate ELISA kits. TLR-4 expression was significantly higher in the PTB (*p* > 0.05) group than in the FTB group. Of the five proinflammatory cytokines, IL-6, IL-7, IL-1β, and TNF-α were significantly higher in the PTB (*p* < 0.05) group, whereas the other cytokine, CCL3, was also higher, but the difference was not significant compared with that in the FTB group (Figure 3).

### 3.5. Correlation Analysis

**Figure 4:**
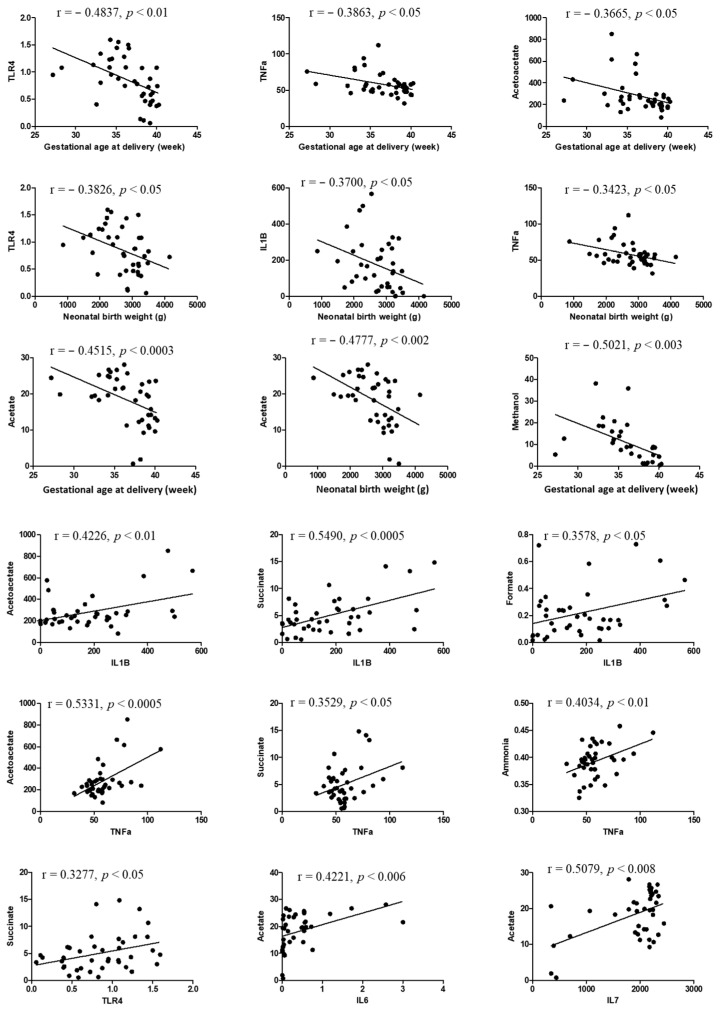

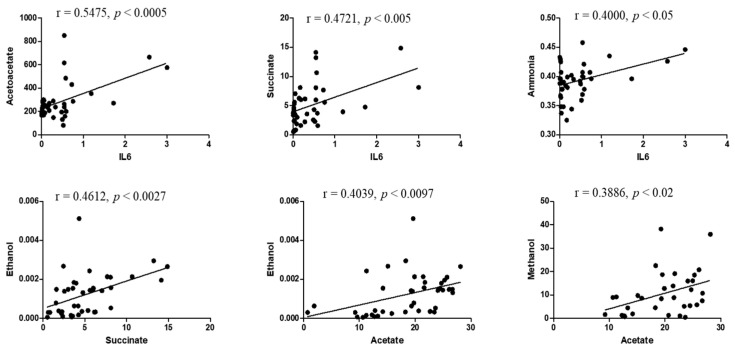
Correlation between clinical and biochemical data. Results of correlation analysis performed among clinical parameters, cytokines, and metabolites. *p* < 0.05 was considered statistically significant.

In our correlation analysis, we found significant correlations between clinical and biochemical data. Gestational age at delivery showed a significant negative correlation with TLR4, TNF-α, and acetoacetate (r = −0.4837, r = −0.3863, and r = −0.3665, respectively) (*p* < 0.05). Furthermore, neonatal body weight showed a significant negative correlation with TLR4, IL-1β, and TNF-α (r = -0.3826, r = −0.3700, and r = −0.3423, respectively) (*p* < 0.05). Metabolites like acetate and methanol showed a significant negative correlation with gestational age at delivery and infant body weight (r = −0.4515, 0.0477, and 0.5021, respectively) (*p* < 0.05). Additionally, a positive correlation was observed between cytokine and metabolite levels. IL-6 was positively correlated with acetoacetate, succinate, and ammonia (r = 0.5475, r = 0.4721, and r = 0.4000, respectively) (*p* < 0.05); IL-1β was positively correlated with acetoacetate, succinate, and formate (r = 0.4226, r = 0.5490, and r = 0.3578, respectively); and TNF-α was positively correlated with acetoacetate, succinate, and ammonia (r = 0.5331, r = 0.3529, and r = 0.4031, respectively) (*p* < 0.05). Furthermore, TLR4 was positively correlated with succinate (r = 0.3277) (*p* < 0.05). Additionally, ethanol significantly positively correlated with succinate and acetate (r = 0.4612 and r = 0.4039, respectively), and methanol positively correlated with acetate (r = 0.3886) (*p* <0.05) (Figure 4).

## 4. Discussion

In this study, we found that DVM induced the PTB cascade via microbiota-generated metabolites and inflammatory markers. DVM disturbs the vaginal environment during pregnancy and may facilitate PTB outcomes [22]. Moreover, DVM is associated with pathogenic metabolites and inflammation of the intrauterine space [2,23]. DVM affects the availability of microbiota metabolites (such as lactic acid and TMAO) and inflammatory markers (such as TLR-4 and IL-6) [5,6]. In our previous studies, we found an association of vaginal and blood microbiota, cytokines, and metabolites with PTB [3,4,19,21]. Therefore, we investigated the association of the microbiota, metabolites, and cytokines with birth outcomes. We found that TLR-4 and TNF-α were negatively correlated with neonatal gestational age at delivery and birth weight and that TLR-4, IL-6, IL-1β, and TNF-α were positively correlated with acetoacetate, succinate, and ammonia.

The dominance of *Lactobacillus* spp. is a biomarker of a healthy vaginal environment and an indicator of FTB [14]. Approximately 20 species of *Lactobacillus* are found in the human vagina, and a high abundance of *L. crispatus*, *L. iners*, *L. jensenii*, and *L. gasseri* is indicative of a healthy vaginal environment [24]. In this study, CVF microbiota profiling revealed that the total relative abundance of *Lactobacillus* spp. (such as *L. gasseri*, and *L. jensenii*) was higher in the FTB group than in the PTB group, suggesting that it is a good indicator of FTB [10]. Additionally, the total relative abundance of *L. crispatus* was higher than that of other *Lactobacillus* spp. in PTB, which has also been previously observed in Korean women (non-pregnant, pregnant, term, and PTB) [25]. The CVF samples belong to the 3rd trimester, which might be because the total relative abundance of *Lactobacillus spp.* in FTB and PTB were not significantly different. Additionally, *Bifidobacterium* may shape healthy full-term pregnancy and neonate development through protection against inflammation, as we also observed a high relative abundance of *Bifidobacterium* in the FTB group [26,27]. The high relative abundances of *Lactobacillus* spp. and *Bifidobacterium* suggest that they are associated with protection against inflammation and PTB.

Several physiological changes, mainly hormonal (estrogen and progesterone levels) and metabolic changes, influence the relative abundance of dysbiotic vaginal microbiota. *Lactobacillus* spp. protect against infections and the invasion of pathogenic microbes and maintain a low pH (4.0 to <4.5), whereas *Lactobacillus* spp. dysbiosis is considered a hallmark of an abnormal vaginal environment [12,13]. However, the low abundance of *Lactobacillus* spp. and the high abundance of anaerobic bacteria, such as *Prevotella*, *Dialister*, *Atopovium*, *Gardnerella*, *Megasphaera*, *Peptoniphilus*, *Sneatia*, *Eggertella*, *Aerococcus*, *Finegoldia*, and *Mobiluncus*, disturb the vaginal environment and pH (>4.5) [10]. A disturbed vaginal environment with an abundance of anaerobic bacteria is associated with an Increased risk of vaginal infection and inflammation, resulting in PTB [11]. High abundances of *Corynebacterium amycolatum*, *Enterococcus faecalis*, *Staphylococcus*, *Prevotella*, *and Ureaplasma parvum* also induce PTB [28]. Furthermore, *Prevotella*, *Gardnerella*, *Ureaplasma parvum*, *Finegoldia*, and *Aerococcus* increase the pH of the vaginal environment compared to an environment with *Lactobacillus* spp. [9,10,29,30]. A high pH (>4.5) is considered the first indication of PTB, as we observed a high pH (>4.5) in the CVF samples of the PTB group, which might be due to the high relative abundance of anaerobic microbiota [8]. In this study, we observed a high relative abundance of *Corynebacterium* spp., which produces ethylene glycol, a pathogenic microbial metabolite that influences PTB [19]. A high diversity of anaerobic pathogenic microbes indicates BV and is associated with an increased risk of vaginal infection, inflammation, and PTB [11,14]. Here, PTB women did not have a vaginal infection like BV but a state of increased anaerobic microbiota without significantly decreased *Lactobacillus* spp. Recently, we found asymptomatic BV improved with the treatment of three *Lactobacillus* spp. combination probiotics [31].

During pregnancy, DVM directly affects the microbiota as well as metabolite production and availability, which disturbs the vaginal environment and affects birth outcomes [4,19]. Microbiota produce metabolites from dietary molecules, such as sugars and proteins, which may be disturbed by microbiota dysbiosis, resulting in accelerated metabolic pathways [32]. With a low abundance of *Lactobacillus* spp. during pregnancy, DVM reflects low L-lactic acid production, which might affect birth outcomes, which is supported by the low L-lactic acid levels observed in PTB in this study [33]. Altered concentrations of metabolites such as short-chain fatty acids (acetate and succinate) can also serve as biomarkers. Previous studies have revealed that altered succinate levels can increase the risk of PTB; consistent with these findings, we observed high succinate levels in the PTB group [34]. The alcoholic metabolite formaldehyde is converted from methanol into formate by bacterial alcohol dehydrogenase. We observed high formate levels in PTB, which are a diagnostic measure of methanol toxicity during pregnancy [35]. Ethanol and methanol are major alcohol toxicants, which are carbohydrate-fermented primary metabolites that can regulate metabolic dysfunction [36]. We observed high ethanol and methanol levels in PTB, which significantly correlated with clinical (gestational age at delivery) and biochemical parameters (succinate and acetate). Furthermore, high levels of the protein metabolite TMAO are found in PTB; this overproduction of TMAO is attributed to the dysmetabolism of choline by the microbiota [19,37]. TMAO is converted into ammonia and formaldehyde in the presence of TMA demethylase, as indicated by significantly high levels of ammonia and formaldehyde in PTB [38]. These disturbed metabolites serve as pathogen-associated molecular patterns (PAMPs) and may trigger inflammatory signaling through TLRs [39].

TLRs are PRRs that recognize PAMPs derived from microorganisms [40]. PAMPs include carbohydrate derivatives, proteins (polypeptides), and nucleic acids that are generated and expressed by microorganisms [41] and induce TLRs as messengers to initiate inflammatory cascades, which in turn result in PTB [42]. In humans, ten TLR family members (TLR1–10) have been identified as crucial for inflammatory immune responses [43]. TLR4, in particular, is a key regulator of the inflammatory process and is abundantly expressed in the placenta, fetal membrane, and uterus [44]. Although TLR4 is a transmembrane receptor, soluble forms of some TLRs have recently been detected in biofluids [45,46]. In the present study, we observed high levels of the soluble form of TLR4 in CVF samples from the PTB group. Furthermore, correlation analysis of TLR4 showed a negative correlation between gestational age at delivery and neonatal birth weight, indicating its potential as an indicator of PTB [42].

TLR4 recognizes PAMPs and activates inflammatory pathways and proinflammatory cytokine production [47]. *Lactobacillus* spp. in the vagina inhibit pathogen invasion via their metabolite, L-lactic acid, and inhibit inflammatory pathways [15,48]. L-lactic acid has anti-inflammatory properties that inhibit the production of proinflammatory cytokines (IL6) and chemokines (CCL3) induced by TLRs in vaginal epithelial cells at low pH (4.0 to <4.5) [15]. High levels of succinate and acetate affect proinflammatory cytokines, resulting in PTB outcomes [16,17]. The chemokine CCL3 is produced by macrophages in response to bacterial products and increases the production of IL6, TNF, and IL1β, as observed in the PTB group [49,50]. The high levels of cytokines were significantly correlated with metabolites, indicating a favorable condition for PTB.

## 5. Conclusions

In conclusion, this multi-approach analysis revealed that DVM alters normal pregnancy through high vaginal pH (>4.5), low abundance of *Lactobacillus* spp., and high TLR4 expression, thus inducing PTB via pathogenic molecules such as microbial metabolites and cytokines in the vagina. The number of cases is small, which is a limitation due to the fact that the study was conducted during COVID-19. Further research with a greater number of subjects is needed to give better outcomes of microbiota metabolites and cytokines correlation and prediction of preterm birth.

## Figures and Tables

**Table 1:** Demographic profile of the subjects and variables analysis.

Variables	FTB (n = 20)	PTB (n = 20)	*p*-Value
Maternal variables at CVF sampling			
Age (yrs)	32.60 ± 0.62	34.42 ± 1.19	NS
Body mass index (kg/m^2^)	21.24 ± 0.58	22.65 ± 1.24	NS
Gestational age at sampling (wks)	34.37 ± 1.8	33.34 ± 0.57	NS
Cervical length (mm)	23.92 ± 1.9	25.04 ± 2.70	NS
Cervicovaginal fluid pH	3.57 ± 0.60	4.57 ± 0.50	0.002
Neonate variables at birth			
Gestational age at delivery (wks)	38.97 ± 0.18	33.96 ± 0.58	<0.0001
Birth weight (gm)	3172 ± 75.68	2240 ± 128.90	<0.0001
1 min Apgar score	9.50 ± 0.17	7.79 ± 0.59	0.0076
5 min Apgar score	9.95 ± 0.05	8.68 ± 0.41	0.0039

FTB, full-term birth; PTB, preterm birth, Apgar score, appearance, pulse, grimace, activity, and respiration. Statistical significance was set at *p* < 0.05. NS: Not significant.

## Data Availability

The original contributions presented in the study are included in the article/Supplementary Material, further inquiries can be directed to the corresponding author/s. The microbiota data presented in this study are available in Supplementary Material.

## Supplementary Materials

The following supporting information can be downloaded at https://www.mdpi.com/article/10.3390/metabo14010045/s1, Supplementary S1 Figure S1. Microbiota profiled in cervicovaginal fluid (CVF) samples of full-term birth (FTB, n = 7) and preterm birth (PTB, n = 7) groups using next-generation sequencing of the V1–V9 region. Supplementary S1 Figure S2. Metabolite analysis in cervicovaginal fluid (CVF) samples. Metabolites measured in CVF samples of full-term birth (FTB, n = 20) and preterm birth (PTB, n = 20) groups using the assay kits. Data are presented as the mean ± standard deviation, and *p* < 0.05 was considered statistically significant; *** *p* < 0.001. Supplementary S2: microbiota data analysis.
