# Supplementary material for: Dysbiotic Vaginal Microbiota Induces Preterm Birth Cascade via Pathogenic Molecules in the Vagina
Source: Metabolites. 2024 Jan 11;14(1):45. doi: 10.3390/metabo14010045 (PMC10821287; doi:10.3390/metabo14010045)
Supplement: Supplementary file 1 [file metabolites-14-00045-s001.zip › metabolites-2774833-supplementary S1 (Figure S1, S2).pdf]

Supplementary Materials

Supplementary Figure S1

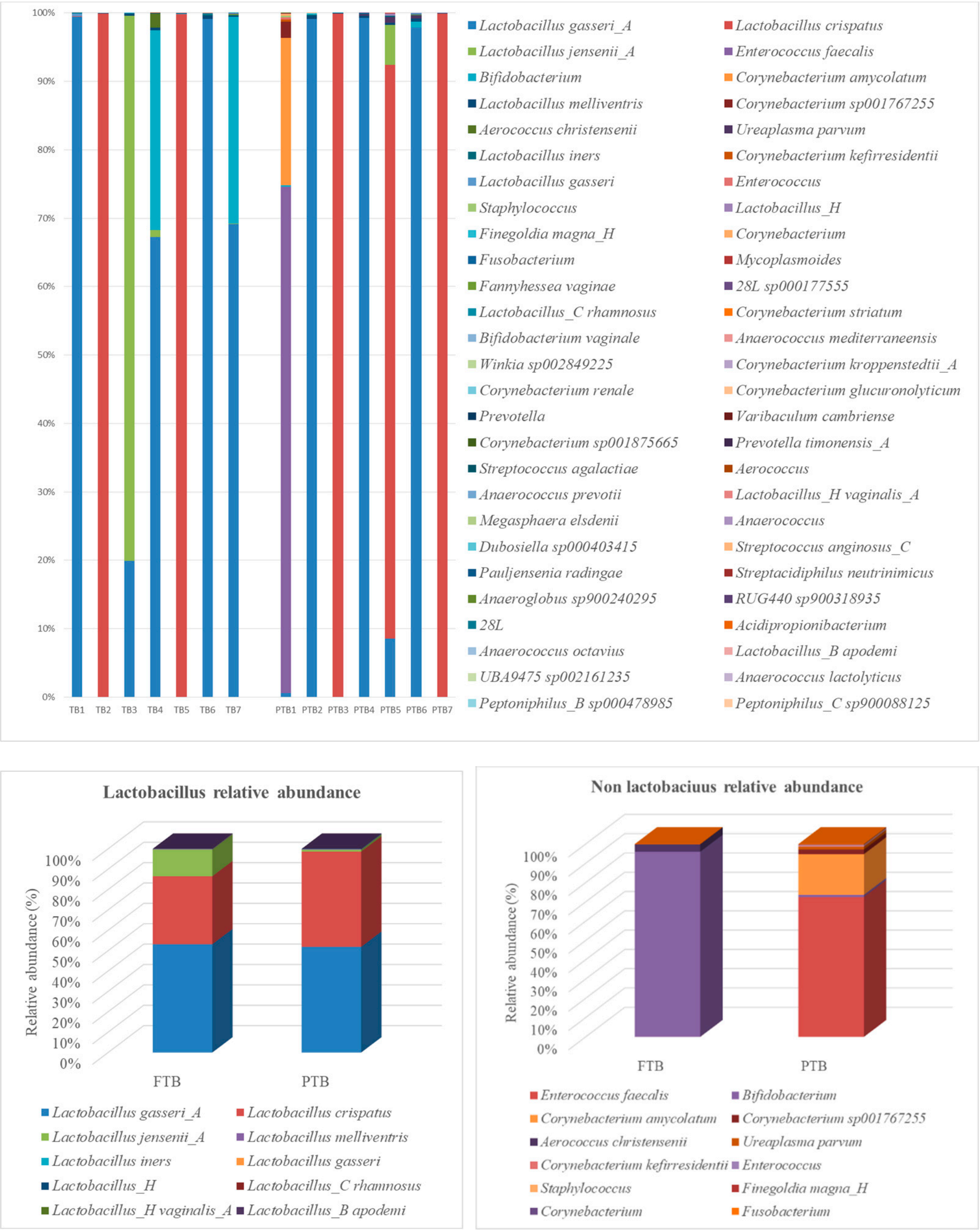

Figure S1. Microbiota profiling of cervicovaginal fluid (CVF) samples of full-term birth (FTB, n=7) and preterm birth (PTB, n=7) groups via next generation sequencing of the V1–V9 region.

Supplementary Figure S2

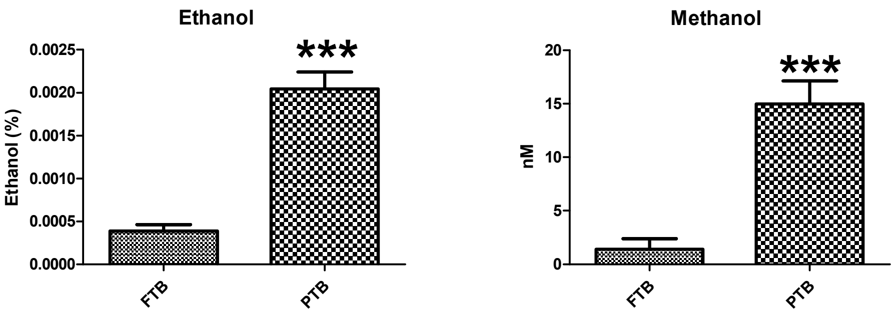

Figure S2. Metabolite analysis in cervicovaginal fluid (CVF) samples. Metabolites measured in CVF samples of full-term birth (FTB, n = 20) and preterm birth (PTB, n = 20) groups using the assay kits. Data are presented as the mean ± standard deviation, and $p < 0.05$ was considered statistically significant; *** $p < 0.001$.
