# Supplementary material for: Dysbiotic Vaginal Microbiota Induces Preterm Birth Cascade via Pathogenic Molecules in the Vagina
Source: Metabolites. 2024 Jan 11;14(1):45. doi: 10.3390/metabo14010045 (PMC10821287; doi:10.3390/metabo14010045)
Supplement: Supplementary file 1 [file metabolites-14-00045-s001.zip › supplementary S2-microbiota data analysis.zip!/DNALink_Metagenome_Service.html]

DNALink\_Metagenome\_Service


Toggle Menu

*Genome Analysis Service*

- **Service Information**


- **Workflow**

- **Results**

2F, Cluster Center,  
     150, Bugahyeon-ro, Seodaemun-gu,   
    Seoul, Republic of Korea
  


 +82.2.3153.1500
  
 +82.2.364.4778


# **Service Information**

Brief information of your service is below. Please check your service information.

Service ID
:   20210622\_EUMC\_LeeGain\_Metagenome\_1

Client
:   Lee\_Gain

Company/Institute
:   EUMC

Service Type
:   Metagenome

Number of Samples
:   14
  

Sequencing Platform
:   PacBio Sequel

Library
:   16S Full Length Library

Basecalling Software
:   SmrtLink v9
  

Demultiplexer
:   ShorelineBiome Analyzer

OTU Clustering Cutoff
:   97%

Taxonomy Database
:   GTDB

Ask Here
