# Supplementary material for: Dysbiotic Vaginal Microbiota Induces Preterm Birth Cascade via Pathogenic Molecules in the Vagina
Source: Metabolites. 2024 Jan 11;14(1):45. doi: 10.3390/metabo14010045 (PMC10821287; doi:10.3390/metabo14010045)
Supplement: Supplementary file 1 [file metabolites-14-00045-s001.zip › supplementary S2-microbiota data analysis.zip!/templatesets/results/beta_diversity/unweighted_unifrac_emperor/index.html]

q2-emperor : plot
