# Supplementary material for: Dysbiotic Vaginal Microbiota Induces Preterm Birth Cascade via Pathogenic Molecules in the Vagina
Source: Metabolites. 2024 Jan 11;14(1):45. doi: 10.3390/metabo14010045 (PMC10821287; doi:10.3390/metabo14010045)
Supplement: Supplementary file 1 [file metabolites-14-00045-s001.zip › supplementary S2-microbiota data analysis.zip!/templatesets/src/otu_results.html]

OTU-DNALink\_Metagenome\_Service


Toggle Menu

*Genome Analysis Service*

- **Service Information**


- **Workflow**

- **Results**

2F, Cluster Center,  
     150, Bugahyeon-ro, Seodaemun-gu,   
    Seoul, Republic of Korea
  


 +82.2.3153.1500
  
 +82.2.364.4778


# **OTU Clustering Results**

Reads are de novo clustered into OTUs(Operational Taxonomic Units) at 97% similarity.

#### **OTU Table Summary**

  

| Sample | # of OTUs | # of Singletons | # of Doubletons | # of Reads |
| --- | --- | --- | --- | --- |
| T1 | 143 | 95 | 13 | 8,882 |
| T10 | 331 | 233 | 32 | 11,368 |
| T11 | 155 | 105 | 19 | 10,626 |
| T12 | 257 | 185 | 25 | 12,379 |
| T13 | 176 | 123 | 18 | 11,591 |
| T14 | 262 | 191 | 20 | 5,630 |
| T15 | 224 | 149 | 23 | 11,933 |
| T16 | 170 | 126 | 10 | 11,193 |
| T2 | 98 | 73 | 6 | 12,458 |
| T3 | 159 | 106 | 19 | 6,665 |
| T5 | 209 | 139 | 28 | 10,201 |
| T6 | 112 | 72 | 16 | 12,155 |
| T7 | 158 | 110 | 19 | 12,308 |
| T9 | 210 | 141 | 22 | 11,761 |

  
  

Operational Taxonomic Units(OTUs)
:   cluster of similar sequence variants of the 16S rDNA marker gene sequence

**Back**

Ask Here
