# Supplementary material for: Dysbiotic Vaginal Microbiota Induces Preterm Birth Cascade via Pathogenic Molecules in the Vagina
Source: Metabolites. 2024 Jan 11;14(1):45. doi: 10.3390/metabo14010045 (PMC10821287; doi:10.3390/metabo14010045)
Supplement: Supplementary file 1 [file metabolites-14-00045-s001.zip › supplementary S2-microbiota data analysis.zip!/templatesets/src/results.html]

Results-DNALink\_Metagenome\_Service


Toggle Menu

*Genome Analysis Service*

- **Service Information**


- **Workflow**

- **Results**

2F, Cluster Center,  
     150, Bugahyeon-ro, Seodaemun-gu,   
    Seoul, Republic of Korea
  


 +82.2.3153.1500
  
 +82.2.364.4778


# **Results**

Results are able to be seen by clicking 'Results' buttons below. You can also download the files with 'Download' buttons.

**Checkpoint**  
If you need ID and Password for the download, please check ID and Password below.
  
**ID                        user1**  
**Password    dnalink1234%**

| Category | Results |
| --- | --- |
| Sequencing Results | Seq\_Results |
| OTU Clustering | OTU\_Results |
| Taxonomy Analysis | Taxon\_Results |
| Alpha Diversity | Alpha\_Results |
| Beta Diversity | Beta\_Results |
| Data Download | Download |

Ask Here
