# Supplementary material for: Dysbiotic Vaginal Microbiota Induces Preterm Birth Cascade via Pathogenic Molecules in the Vagina
Source: Metabolites. 2024 Jan 11;14(1):45. doi: 10.3390/metabo14010045 (PMC10821287; doi:10.3390/metabo14010045)
Supplement: Supplementary file 1 [file metabolites-14-00045-s001.zip › supplementary S2-microbiota data analysis.zip!/templatesets/src/beta_results.html]

BetaDiversity-DNALink\_Metagenome\_Service


Toggle Menu

*Genome Analysis Service*

- **Service Information**


- **Workflow**

- **Results**

2F, Cluster Center,  
     150, Bugahyeon-ro, Seodaemun-gu,   
    Seoul, Republic of Korea
  


 +82.2.3153.1500
  
 +82.2.364.4778


# **Beta Diversity Results**

Unweighted UniFrac, Weighted UniFrac, Jaccard, and Bray-Curtis PCoA(Principal Coordinates Analysis) Plots.

#### **Beta Diversity Indices**

  

| Index | Results |
| --- | --- |
| Unweighted UniFrac | Unweighted\_UniFrac\_PCoA |
| Weighted UniFrac | Weighted\_UniFrac\_PCoA |
| Bray-Curtis | Bray\_Curtis\_PCoA |
| Jaccard | Jaccard\_PCoA |

  
  

Beta Diversity
:   ratio between regional and local species diversity
  

UniFrac Distance
:   measures the distance between communities based on the lineages they contain  
        weighted (quantitative) and unweighted (qualitative)

Bray-Curits Distance
:   quantify the compositional dissimilarity between two different sites, based on counts at each site

Jaccard Distance
:   a statistic used for gauging the similarity and diversity of sample sets

**Back**

Ask Here
