# Supplementary material for: Dysbiotic Vaginal Microbiota Induces Preterm Birth Cascade via Pathogenic Molecules in the Vagina
Source: Metabolites. 2024 Jan 11;14(1):45. doi: 10.3390/metabo14010045 (PMC10821287; doi:10.3390/metabo14010045)
Supplement: Supplementary file 1 [file metabolites-14-00045-s001.zip › supplementary S2-microbiota data analysis.zip!/templatesets/src/alpha_results.html]

AlphaDiversity-DNALink\_Metagenome\_Service


Toggle Menu

*Genome Analysis Service*

- **Service Information**


- **Workflow**

- **Results**

2F, Cluster Center,  
     150, Bugahyeon-ro, Seodaemun-gu,   
    Seoul, Republic of Korea
  


 +82.2.3153.1500
  
 +82.2.364.4778


# **Alpha Diversity Results**

In this page, you can see the alpha diversity index values and rarefaction curve plot.

| Category | Results |
| --- | --- |
| Alpha Rarefaction Curve | Alpha\_Rarefaction\_Curve |
| Alpha Diversity Index Values | Alpha\_Index |

  
  

Alpha Rarefaction Curve
:   Curve plot of observed number of OTUs as a function of the number of resampled sequences in the OTU table

**Back**

Ask Here
