# Supplementary material for: Dysbiotic Vaginal Microbiota Induces Preterm Birth Cascade via Pathogenic Molecules in the Vagina
Source: Metabolites. 2024 Jan 11;14(1):45. doi: 10.3390/metabo14010045 (PMC10821287; doi:10.3390/metabo14010045)
Supplement: Supplementary file 1 [file metabolites-14-00045-s001.zip › supplementary S2-microbiota data analysis.zip!/templatesets/src/alpha_index.html]

AlaphIndex-DNALink\_Metagenome\_Service


Toggle Menu

*Genome Analysis Service*

- **Service Information**


- **Workflow**

- **Results**

2F, Cluster Center,  
     150, Bugahyeon-ro, Seodaemun-gu,   
    Seoul, Republic of Korea
  


 +82.2.3153.1500
  
 +82.2.364.4778


# **Alpha Diversity Results**

ACE, Chao1, Evenness, Faith\_PD, Good's Coverage, Observed OTUs, Shannon, and Simpson values

| Sample | Chao1 | Evenness | Faith PD | Good's Coverage | Observed OTUs | Shannon | Simpson |
| --- | --- | --- | --- | --- | --- | --- | --- |
| T1 | 245.188 | 0.293 | 8.595 | 0.988 | 107 | 1.976 | 0.615 |
| T10 | 856.000 | 0.332 | 59.876 | 0.973 | 210 | 2.563 | 0.712 |
| T11 | 251.176 | 0.164 | 15.850 | 0.987 | 105 | 1.098 | 0.356 |
| T12 | 387.056 | 0.205 | 38.213 | 0.983 | 139 | 1.460 | 0.428 |
| T13 | 437.667 | 0.169 | 31.252 | 0.986 | 104 | 1.130 | 0.371 |
| T14 | 1,126.048 | 0.330 | 22.575 | 0.966 | 262 | 2.649 | 0.635 |
| T15 | 471.176 | 0.198 | 32.002 | 0.981 | 150 | 1.435 | 0.426 |
| T16 | 439.545 | 0.186 | 27.709 | 0.985 | 115 | 1.276 | 0.412 |
| T2 | 274.250 | 0.174 | 6.400 | 0.993 | 59 | 1.024 | 0.371 |
| T3 | 366.714 | 0.301 | 14.459 | 0.983 | 145 | 2.161 | 0.639 |
| T5 | 329.043 | 0.276 | 10.349 | 0.983 | 139 | 1.966 | 0.612 |
| T6 | 313.500 | 0.185 | 11.085 | 0.992 | 66 | 1.119 | 0.406 |
| T7 | 309.250 | 0.178 | 13.425 | 0.989 | 88 | 1.148 | 0.367 |
| T9 | 538.909 | 0.352 | 11.487 | 0.983 | 133 | 2.487 | 0.736 |

  
  

ACE(Abundance-based Coverage Estimator)
:   based on the concept of "sample coverage"

Chao1
:   how many species are represented by only one individual in the sample (singletons), and how many species are represented by exactly two individuals (doubletons)

Evenness
:   measure of the relative abundance of the different species making up the richness of an area

Faith\_PD
:   measure of biodiversity, based on phylogeny (the tree of life)

Good's Coverage
:   estimating what percent of the total species is represented in a sample

Observed\_OTUs
:   measure of community richness

Shannon
:   accounting for both abundance and evenness of the species present.

Simpson
:   measure of the probability that two individuals randomly selected from a sample will belong to the same species

**Back**

Ask Here
