# Supplementary material for: Dysbiotic Vaginal Microbiota Induces Preterm Birth Cascade via Pathogenic Molecules in the Vagina
Source: Metabolites. 2024 Jan 11;14(1):45. doi: 10.3390/metabo14010045 (PMC10821287; doi:10.3390/metabo14010045)
Supplement: Supplementary file 1 [file metabolites-14-00045-s001.zip › supplementary S2-microbiota data analysis.zip!/templatesets/src/workflow.html]

Workflow-DNALink\_Metagenome\_Service


Toggle Menu

*Genome Analysis Service*

- **Service Information**


- **Workflow**

- **Results**

2F, Cluster Center,  
     150, Bugahyeon-ro, Seodaemun-gu,   
    Seoul, Republic of Korea
  


 +82.2.3153.1500
  
 +82.2.364.4778


# **Workflow**

The anslysis is based on **QIIME2(Quantitative Insights Into Microbial Ecology 2)**. See more details here.

Image from https://docs.qiime2.org/2020.8/tutorials/overview/#let-s-get-oriented-flowcharts

  


Ask Here
