# Supplementary material for: Dysbiotic Vaginal Microbiota Induces Preterm Birth Cascade via Pathogenic Molecules in the Vagina
Source: Metabolites. 2024 Jan 11;14(1):45. doi: 10.3390/metabo14010045 (PMC10821287; doi:10.3390/metabo14010045)
Supplement: Supplementary file 1 [file metabolites-14-00045-s001.zip › supplementary S2-microbiota data analysis.zip!/templatesets/src/seq_results.html]

SequencingResults-DNALink\_Metagenome\_Service


Toggle Menu

*Genome Analysis Service*

- **Service Information**


- **Workflow**

- **Results**

2F, Cluster Center,  
     150, Bugahyeon-ro, Seodaemun-gu,   
    Seoul, Republic of Korea
  


 +82.2.3153.1500
  
 +82.2.364.4778


# **Sequencing Results**

Check the sequencing results of samples.

#### **Demultiplexed Data Summary**

| Sample | # of Bases | # of Reads | N50 Read Length | Mean Read Length |
| --- | --- | --- | --- | --- |
| T1 | 68,328,098 | 10,256 | 962,174 | 6,662 |
| T10 | 84,384,652 | 12,714 | 1,730,834 | 6,637 |
| T11 | 74,809,824 | 11,420 | 1,084,916 | 6,550 |
| T12 | 82,735,096 | 13,115 | 1,546,545 | 6,308 |
| T13 | 79,788,281 | 12,264 | 765,345 | 6,505 |
| T14 | 45,342,758 | 7,362 | 1,338,971 | 6,159 |
| T15 | 84,068,748 | 12,772 | 1,052,737 | 6,582 |
| T16 | 75,622,081 | 11,870 | 1,144,981 | 6,370 |
| T2 | 85,110,637 | 13,205 | 1,004,942 | 6,445 |
| T3 | 52,975,204 | 8,342 | 1,364,396 | 6,350 |
| T5 | 75,760,179 | 11,486 | 1,240,527 | 6,595 |
| T6 | 83,984,721 | 12,841 | 1,035,322 | 6,540 |
| T7 | 85,980,328 | 13,009 | 1,149,272 | 6,609 |
| T9 | 86,144,778 | 13,128 | 1,390,028 | 6,561 |

  
  
  

**Back**

Ask Here
